# Decentering as a core component in the psychological treatment and prevention of youth anxiety and depression: a narrative review and insight report

**DOI:** 10.1038/s41398-021-01397-5

**Published:** 2021-05-14

**Authors:** Marc P. Bennett, Rachel Knight, Shivam Patel, Tierney So, Darren Dunning, Thorsten Barnhofer, Patrick Smith, Willem Kuyken, Tamsin Ford, Tim Dalgleish

**Affiliations:** 1 Medical Research Council Cognition and Brain Sciences Unit, University of Cambridge, Cambridge, UK; 2 School of Psychology, University of Surrey, Guildford, UK; 3 Institute of Psychiatry, Kings College London, London, UK; 4 Department of Psychiatry, University of Oxford, Oxford, UK; 5 Department of Psychiatry, University of Cambridge, Cambridge, UK; 6 Cambridgeshire and Peterborough NHS Foundation Trust, Cambridge, UK

**Keywords:** Depression, Scientific community

## Abstract

Decentering is a ubiquitous therapeutic concept featuring in multiple schools of psychological intervention and science. It describes an ability to notice to day-to-day psychological stressors (negative thoughts, feelings, and memories) from an objective self-perspective and without perseverating on the themes they represent. Thus, decentering dampens the impact and distress associated with psychological stressors that can otherwise increase mental ill health in vulnerable individuals. Importantly, the strengthening of decentering-related abilities has been flagged as a core component of psychological interventions that treat and prevent anxiety and depression. We provide an in-depth review evidence of the salutary effects of decentering with a special focus on youth mental health. This is because adolescence is a critical window for the development of psychopathology but is often under-represented in this research line. A narrative synthesis is presented that integrates and summarizes findings on a range of decentering-related abilities. Section 1 reviews extant conceptualizations of decentering and data-driven approaches to characterize its characteristic. A novel definition is then offered to guide future empirical research. Section 2 overviews laboratory-based research into the development of decentering as well as its relationship with anxiety and depression. Section 3 examines the role decentering-related skills play in psychological interventions for anxiety and depression. Critically, we review evidence that treatment-related increases in decentering predict latter reductions in anxiety and depression severity. Each section highlights important areas for future research. The report concludes by addressing the vital questions of whether, how, why and when decentering alleviates youth anxiety and depression.

## Introduction

People reflect on their feelings, memories, and thoughts – especially emotional ones – to abstract meaning about themselves. This practice of mentalizing emerges early in life^1,2^ and it can promote either psychological well-being or mental ill-health^3^. An example of adaptive self-observation is to notice, not only the negative content of inner events, but also the cognitive activities that generate them. This self-observation technique – referred to as decentering or decentering-related abilities – allows individuals to experience distressing inner events as imperfect models of the real-world rather than precise reflections. Adopting this objective self-perspective can thus prevent inner events from disproportionately influencing affect, behavior and sense-of-self. For instance, a person might notice “I am thinking I am depressed right now” instead of only noticing and then believing the thought “I am depressed”^4^. Decentering is therefore diametric to more maladaptive self-observation styles e.g. a tendency to view inner events, and the themes they represent, as genuine and immutable facts.

Therapeutic techniques that target decentering are ubiquitous across psychological therapy and science. In fact, the strengthening of decentering-related abilities has long been highlighted as a core component through which multiple interventions accrue positive mental health outcomes^5,6^. This review integrates evidence from different approaches to determine whether, how, why and when decentering-related abilities alleviate youth anxiety and depression. Findings are based on a review of peer-reviewed studies of decentering and related constructs (for methods, see Supplemental Materials). We present a narrative synthesis of the literature across three sections that: (1) characterize and define decentering; (2) chart the development of decentering, and its relationship with anxiety and depression; and (3) examine the mediating role of decentering in evidence-based psychological interventions. The clinical implications of decentering and directions for further research are highlight throughout these sections (also, see Supplemental Box 1). Furthermore, insights on decentering from a panel of youth advisors is outlined in Supplemental Boxes 2–5.

## Foundations of decentering

### Defining and measuring decentering

Different terms describe an ability to notice negative inner events from an objective perspective without excessively and inappropriately reacting to their content (Table 1)^7^. Within mindfulness-based treatments, decentering is an ability to observe thoughts/feelings as temporary events in the mind rather than true reflections of reality and the self^8^. This cognitive set may reduce the tendency to treat inner events like genuine realities and dampen accompanying distress. Detached mindfulness is the ability to detach from thoughts by observing them from an objective perspective^9^. This entails a meta-cognitive processing mode wherein thoughts are interpreted as transient mental events. Reperceiving is also a meta-cognitive skill characterized by an ability to shift one’s perspective and dis-identify from the content of negative inner events^10^. Likewise, meta-cognitive awareness is referred to as a cognitive processing mode in which thoughts/memories are interpreted with the knowledge that they are transient mental events rather than defining characteristics of the self or reality – this disengagement from the distressing content of inner events undermines their influence over emotion and behavior. Within acceptance-based treatments, cognitive defusion – originally called comprehensive distancing^11^ – describes a way of responding to negative thoughts that entails disengaging from their literal meaning^12,13^. This is achieved through exercises that putatively disrupt verbal processes that transfer meaning between inner events (like thoughts) and their referents (like events)^14–17^. Acceptance-based treatments also refer to the self-as-context, or observer-self perspective, as a distanced locus from which the ‘self’ is discriminated from the flow of thoughts, feelings and memories it encounters. Similarly, the salutary effects of processing negative inner events from a third-person perspective, rather than from an ego-centric perspective, is emphasized in Cognitive Behavioral Therapy (CBT). This is called self-distancing^18^ and it is shaped though exercises that place ‘psychological distance’ between an individual and their inner events, thus allowing individuals to reconstrue negative experiences within a broader context^19,20^.

**Table 1:** Characteristics, measurements, and mechanisms underlying decentering-related abilities.

Term	Functional characteristics	Latent factor^a^	Negative inner events^b^	Common measures	Hypothetical mechanisms (HM)	Theory/model (references)^c^	Intervention
Decentering	Shift in experiential perspective Adopting an objective self-observation style	Observer perspective	Thoughts–memories Feelings	-EQ^d^ -TMS^d^ -MACAM	Meta-awareness reduces distress by facilitating: [HM1] disidentification from negative events. [HM2] new memories of negative events as transient.	[HM1] Meta-cognitive processes model^7^ [HM2] Differential activation hypothesis (52)	MBCT MT
Cognitive defusion	Reduced distress and believability of inner events Reduced influence of thoughts over behavior	Reduced struggle	Thoughts –feelings	-CFQ -DDS -BAFT -Y-AFQ^e^ -AAQ	Defusion reduces distress by disrupting the verbal processes that transfer literal meaning between conceptually related events, such as thoughts and their referent events.	Relational frame theory^17,107,108^	ACT
Self-distancing	‘Stepping back’ from past events Thinking about things from the perspective of a distant observer	Observer perspective	Thoughts –memories	-TDQ -Single item (7-point Likert) e.g. *I saw events through my own eyes -v- I saw events unfold as an observer*.	Self-distancing reduces distress by: [i] introducing ‘*psychological distance*’ between the self and the negative event (e.g. spatial, temporal, objective or hypothetical distance). [ii] this distance allows individuals to focus on broader contexts and to reconstrue their negative experiences.	Construal level theory^19,42,105,106,109,110^	CBT
Self-as-context	Awareness of the flow of inner events without attachment to them	Observer perspective	Thoughts–feelings Memories	-SACS	Self-as-context reduces distress by establishing alternative verbal associations between one’s ‘self concept’ and the inner events it encounters (deictic relational framing).	Relational frame theory^107,111–113^	ACT
Reperceiving	Disidentification from content of inner events Objective awareness of moment-to-moment experiences	Observer perspective	Feelings Thoughts –memories	–	Reperceiving fosters other processes, namely: [i] the self-regulation [ii] cognitive and emotional flexibility [iii] values clarification [iv] and exposure.	Intention, attention, and attitude model of mindfulness^10^	MBCT MT

This table presents a brief overview of the characteristics, measurements, and mechanisms-of-change associated with five popular decentering-related abilities. While this is not an exhaustive list, these particular terms featured prominently in our literature review.

*HM* hypothetical Mechanism, *EQ* experiences questionnaire^4^, *TMS* Toronto mindfulness scale^114^, *MACAM* measure of awareness and coping in autobiographical memory^115^, *CFQ* cognitive fusion questionnaire^36^, *DDS* drexel defusion scale^27^, *BAFT* believability of anxious feelings and thoughts scale^31^, *Y-AFQ* youth action and fusion questionnaire^37^, *AAQ* acceptance and action questionnaire^116^, *TDQ* temporal distancing questionnaire^28^, *SACS* self-as-context scale^32^, *CBT* cognitive behavioral therapy, *MBCT* mindfulness-based cognitive therapy, *MT* mindfulness training, *ACT* acceptance and commitment therapy.

^a^Two factors are reported to underlie decentering-related abilities; these are labeled Observer Perspective and Reduced Struggle (Section 2.2). It has been suggested that different conceptualizations of decentering can emphasis one factor over the other (19).

^b^Decentering-related abilities can be described within the context of specific negative inner events. This row presents an overview of those most commonly associated with specific decentering-related abilities.

^c^Hypothetical mechanisms that to explain the impact of decentering-related abilities on negative affect are often grounded in particular theories/frameworks.

^d^These inventories contain a specific sub-scale for decentering.

^e^To the best of our knowledge, this is the only self-report inventory of a decentering-related ability validated for children and adolescents (Section 3.2).

These therapeutic constructs broadly overlap but are not necessarily inter-changeable. Scientific traditions focus on particular characteristics when describing decentering, e.g., a shift in self-perspective versus a change in emotional reactivity. Constructs can also refer to discrete hypothetical pathways when accounting for the impact decentering on outcomes like emotional state, e.g., change in how self-relevant information is represented in memory versus disruption of verbal processing (Table 1). Such pre-experimental assumptions about the phenomenology of decentering can complicate scientific progress. Specifically, the reliable measurement of decentering is difficult because assessments from different approaches target different features. The Experiences Questionnaire (EQ; the mostly widely used self-report assessment of decentering) correlates only modestly with other inventories including the decentering sub-scale of the Toronto Mindfulness Scale (TMS) (*r* = 0.25) and the Meta-cognitive Awareness Scale (MAS) (*r* = −0.16) (M ± SD = 23.81 ± 4.06 years)^21^. In contrast, EQ scores correlate more strongly with the decentering sub-scale of the Questionnaire on Self-Transcendence (*r* = 0.68−0.77)^22^ as well as self-rated inventories of cognitive defusion, although not to the level one would expect if they were capturing the exact same trait. EQ scores are moderately strongly associated with the Drexel Defusion Scale (DDS) (*r* = 0.39–0.52), Cognitive Fusion Questionnaire (CFQ) (*r* = −0.40 to −0.61) and Acceptance and Action Questionnaire (AAQ) (*r* = −0.67) (19.10–39.57 years)^21,23–27^. EQ scores and the self-rated ability to observe negative inner events from a distanced self-perspective are also moderately associated (*r* = 0.49) (20.7 ± 2.56 years)^28^. The lack of a common model and measure of decentering represents a clear barrier to the discovery and synthesis of new information.

There is a need to develop working definitions of decentering, which can be shared across different scientific traditions, and testable theoretical frameworks. A recent example is the meta-cognitive model of decentering^7,21^. This posits that decentering is a multi-faceted construct involving: [1] the disidentification from negative inner experiences, i.e., an ability to separate one’s self from the content of inner events; and [2] a diminished emotional reactivity to inner events. Furthermore, the mechanism that facilitates disidentification and reduced reactivity is [3] a heightened meta-awareness of underlying cognitive activities. Another example is a recent neurocognitive account of self-distancing – a popular decentering technique in CBT^20,29,30^. This model suggests that a series of neurocognitive processes is implemented by discrete neural regions so to reduce negative emotional states. This begins with a regulatory goal (i.e. feeling less distress), formulated and maintained in working memory, and supported by the dorsolateral prefrontal cortex, anterior cingulate cortex and the pre-sensory motor area. Individuals then engage in affective self-reflection, supported by the dorsomedial prefrontal cortex, to recognize problematic emotional states and stressors. A complex process of self-projection then starts wherein individuals mentally simulate themselves in an alternative space or shifted perspective. This calls on earlier memories and knowledge, supported by the medial temporal lobe, and a capacity to manipulate self-relevant information, supported by the temporal parietal junction. Any change in the initial emotional state is tracked via affective self-reflection so to monitor progress towards the regulatory goal. Meanwhile, new emotional states become possible as individuals perceive surrounding events from a new self-perspective, and this is implemented by areas including the amygdala and ventromedial prefrontal cortex. These models clearly formulate decentering-related abilities in the context of established neuro-cognitive mechanisms. However, there is a paucity of empirical research examining these neuro-cognitive accounts^21,30^.

### Characterizing and re-defining decentering

There have been data-driven attempts to extract key decentering characteristics. In adolescents (19.10 ± 1.60 years), factor analysis indicated two components underlying decentering and cognitive defusion inventories^24^. The first factor was labeled Observer Perspective. This involved an ability to discriminate the self from the inner events it encounters (it included both decentering and cognitive defusion items)^24^. The second factor was labeled Reduced Struggle. This involved dampened emotional reactivity towards negative content of these inner events (it included cognitive defusion items only)^24^. This bivariate structure underpinning decentering-related measures is reported elsewhere. In emerging adults (23.81 ± 4.06 years), two factors explained responses on decentering, meta-cognitive awareness^31^, and cognitive defusion inventories^21^. The first was labeled Intentional Decentered Perspective. This captured the ability to adopt a dis-engaged and non-reactive perspective towards inner events; this resembles the Observer Perspective factor. The second factor was labeled Automatic Reactivity to Thought Content. This reflected heightened emotional reactivity to the negative content of thoughts; an inverse of Reduced Struggle. Similar factors have been reported within the Self-As-Context Scale (SACS) and EQ^32,33^. A reanalysis of the EQ in adult experiencing pain-related distress revealed two factors (47.3 ± 11.69 years)^34^. These were labeled Self-as-Context and Cognitive Fusion (cognitive fusion is simply the inverse of cognitive defusion). The former captured an ability to discriminate one’s self from the flow of negative thoughts it encounters – resembling the Observer Perspective factor. The latter reflected a reduced emotional reactivity to negative thoughts – resembling the Reduced Struggle factor.

These findings suggest two facets underlying measures decentering. The first is an ability to intentionally disengage from the content of inner events by shifting one’s experiential perspective. The second relates to the specific impact of this shift in self-perspective on negative affect; namely, the reduced emotional reactivity to the content of negative inner events. Therefore, we might introduce a novel definition of decentering in the context of negative inner events and emotional states: decentering involves an observed change in emotional reactivity towards negative inner events (like distressing thoughts, memories and feelings) that is associated with a self-reported shift in one’s awareness – away from the thematic content of those events – towards the cognitive activities underlying these events. This working definition effectively operationalizes decentering as it communicates specific and measurable characteristics that can be examined across different scientific and therapeutic traditions. This could also guide new inventories as well as fundamental laboratory research into decentering and its neuro-cognitive correlates. Importantly, this descriptive definition is also agnostic in terms of underlying cognitive mechanism. Definitions based on functional characteristic, rather than a hypothetical mechanism of change, promote better integration of different psychological traditions^35^. This approach also adheres to a fundamental scientific principle: the phenomenon that needs to be explained (explanandum; i.e. decentering) ought to be defined independently from the mechanism used to explain it (explanans; e.g. meta-awareness).

## Decentering, development, and mental health

### The emergence and neuro-cognitive basis of decentering

Evidence suggests that the ability to decenter from everyday negative inner events is continuously distributed^4,28,32,36,37^. Those at the lower end of this continuum are more likely to focus on the thematic content of negative inner events and rely on this information to guide behavior and decision-making^4,36^. That is, inner events are treated as precise models of the real-world. Individuals situated at the higher end of this continuum are instead likely to disengage from negative content by adopting an objective self-perspective^4,36^. That is, inner events are experienced with a greater awareness of their cognitive underpinnings. There are few investigations charting when, and how, this trait emerges. However, laboratory studies indicate that children as young as 4 years can recruit decentering-related abilities to down-regulate negative emotional states^38,39^.

Across three studies, 4–6 year olds but not 3 year olds showed improved behavioral performance^39,40^ and less frustration^41^ during a difficult cognitive task once a decentered self-perspective was adopted (42.51–71.70 months). Decentering was prompted via instructions to adopt a third-person perspective (e.g. talking aloud using their first-name when finding the task stressful) or an objective perspective (e.g. pretending they are someone who would be good at problem-solving like Batman) during frustrating moments of the task. Other studies examined if children and adolescents can decenter from distressing self-relevant events such as negative memories. Here, 10–11 year olds were instructed to recall a fight from the point-of-view of a neutral observer – a self-distanced perspective^42^. This lessened negative feelings and physical reactions relative to those who recalled the conflict as if they were reliving it – a self-immersed perspective.

Evidence also points to the spontaneous use of decentering to reduce negative affect during childhood and adolescence. One study asked 10–20 year olds to verbalize their thoughts and feelings while viewing aversive images^43^. Self-distancing language increased in those who actively attempted to down-regulate emotional response. This was characterized by the less frequent use of first-person singular pronouns like I/me. Importantly, increases in self-distancing language also predicted lower self-rated psychological distress. Another study first instructed 10–17 years old participants to vividly imagine a specific worry about the future^38^. They then rated the extent to which they imagined this event from a self-immersed (‘as if watching things through their own eye’) or self-distanced perspective (‘as if watching themselves in a movie’). Individual differences in decentering were observed with higher self-distance ratings predicting less intense emotional reactivity.

These findings suggest that decentering is an early emergent skill that attenuates emotional reactivity towards negative inner events. This is evident from early childhood studies in which decentering was used spontaneously or because of direct instructions. A small number of studies additionally report that this ability increases over time^38,43,44^, likely due to age-related improvements in executive functioning^41^. Relatedly, some have attempted to elucidate the neuro-cognitive mechanisms underlying the impact of decentering on emotion. Adults are typically instructed to decenter from negative psychological stressors with simultaneous recording of fMRI. Findings suggest lower activity in areas associated with (intrapersonal) cognitive conflict and affective processing when decentering from negative memories relative to a comparison condition in which no attempt was made to alter ones experiences – brain regions included the medial and ventrolateral prefrontal cortex as well as the subgenual anterior cingulate^45^. Also, increased activity in areas associated with perspective taking predicted lower negative affect when decentering relative to the comparison condition – this included the superior parietal lobule, caudate and medial frontal gyrus. Increased activity in areas associated with perspective taking and social perception is also reported when individuals practiced self-distancing from negative images^46^. This included the posterior cingulate and temporal gyrus. However, few have explored the neuro-cognitive processes that mediate the impact of decentering based techniques on negative emotional states^29,30^. Such research may account for individual differences documented in decentering research and present ways optimize extant techniques within psychological interventions.

### The association between decentering and mental health

Those with poor decentering-related abilities are at risk of increased anxiety and depression and lower psycho-social functioning. The earliest reported evidence for this association is during adolescence using the Youth Avoidance and Fusion Questionnaire (Y-AFQ) – a self-report assessment of trait cognitive fusion and affective responding towards distressing inner events. Here, higher scores equate to poorer decentering-related abilities. There is a moderate-strong association between Y-AFQ scores and adolescent anxiety (*r* = 0.49–0.69); that is, anxiety severity increases with the tendency to reify the content of negative inner events^37,44,47^ (12–17 years). A medium–strong association between Y-AFQ scores and adolescent depression is also reported (*r* = 0.55–0.75)^44,47,48^ (12–16 years). Similarly, a lower ability to adopt a ‘self-as-context’ or ‘observer’ perspective towards inner events is associated with increased internalizing difficulties (*r* = −0.476) (15.69 ± 0.56 years)^49^. This was estimated using the Self-As-Context Scale (SACS).

The Y-AFQ is almost exclusively used in adolescent research (12–16 years) while a range of inventories exist for research in older cohorts. Nevertheless, the negative association between decentering-related abilities and symptoms severity persists across development. During later adolescence (~19 years), lower self-rated decentering is associated with increased anxiety, distress and depression. This has been reported in studies using the EQ (*r* anxiety = −0.39) (*r* depression = −0.21 to −0.41)^4,33,50^, the SACS (*r* distress = −0.34)^49^ and TDS (*r* distress = −0.45) (*r* depression = −0.20) (19–21 years)^28^. This negative association is also reported during adulthood using the EQ (*r* anxiety = −0.33 to −0.39; *r* depression = −0.39 to −0.4)^23,26^ and CFQ (*r* anxiety = 0.53–0.64) (*r* depression = −0.45 to −0.61)^25,36,51^ (higher CFQ = poorer decentering). The distancing sub-scale of the recently developed Questionnaire on Self-Transcendence also revealed a negative association between self-rated distancing ability and anxiety and depression symptoms in both adolescents (18.89 ± 1.90 years; *r* anxiety = −0.30; *r* depression = −0.42) and adults (35.21 ± 11.06 years; *r* anxiety = −0.3; *r* depression = −0.48). Additionally, lower self-rated decentering is associated with poorer psycho-social functioning. This is reported across the lifespan: during earlier adolescence via cognitive fusion measures (Y-AFQ, CFQ; *r* = −0.63 to −0.64) (12–13 years)^23,48^; during late adolescence via self-distancing measures (TMDS; *r* = 0.28) and self-as-context (SACS) (*r* = 0.42) (~21 years)^28,32^; and during adulthood via cognitive fusion measures (CFQ) (*r* = 0.21–0.45) (35–43 years)^36,51^.

### Decentering attenuates the impact of negative inner events

Decentering is clearly associated with anxiety and depression from an early age. A key question is – how are they related? One possibility is that an ability to decenter actively protects individuals against, and promotes recovery from, anxiety and depression symptoms^7,52–54^. This is because decentering attenuates the impact and distress associated with day-to-day psychological stressors, which otherwise increase the risk of anxiety and depression onset and maintenance in vulnerable individuals. Such stressors involve inner events like critical self-relevant thoughts or unpleasant memories or feelings^55^. The evidence for this pathway is three-fold.

First, laboratory-based experimental studies with children and adolescents indicate that adopting a decentered perspective (e.g. instructions to adopt a third-person perspective or re-perceive events ‘as if watching themselves in a movie’) dampens emotional reactivity towards aversive stimuli. Stimuli include unpleasant images, video clips or self-relevant statements while emotion change is proxied by decreased negative affect ratings^43,56–58^, reduced believability of inner events^59–63^, lower aggressive behaviors^64^ and shorter emotional episodes^65^. Second, cross-sectional studies indicate that the positive association between psychological stressors and distress is lessened as decentering-related abilities increase. These stressors include negative feelings^66,67^, ruminative thinking^23,34^, early emotional memories^68^, and physical pain^34^. For example, one study indicated that the positive association between unpleasant feelings and depression severity grew weaker as self-rated decentering increased (~19 years)^67^. Increased decentering also dampened the positive association between unpleasant feelings and anxiety severity^67^. Third, improvements in decentering-related abilities that are accrued during psychological interventions predict reduced anxiety and depression^69–71^ (section 4).

Future research should characterize the relationship between decentering and mental health starting from early childhood. Longitudinal studies are required to establish: [1] whether improved decentering-related abilities predict future resilience against anxiety and depression, thus establishing whether decentering is a protective skill in mental health: and [2] when the spontaneous use of decentering begins to impact affect, thus identifying critical periods for training and intervention. These issues remain unexplored since much research takes place after the typical age-of-onset for anxiety and depression. Such investigations would also benefit from reliable and child/adolescent friendly measurements of decentering (e.g. youth variants of the EQ or CFQ) as well as more objective, performance-based tests of decentering-related skills^72^.

## Decentering in psychological interventions

### The reinforcement of decentering

Decentering is a malleable ability that is strengthened in a myriad of psychological interventions (Table 2). In adults seeking treatment for anxiety and depression, medium-large increases in self-rated decentering are reported following CBT (*d* = 0.7–0.82) (~43 years)^52,73^, mindfulness-based interventions (*d* = 0.6–1.01) (34.49–53.30 years)^26,52,74–76^ and acceptance-based therapies (*d* = 0.32–1.85) (37.00–46.40 years)^73,77–80^. Increased self-rated decentering is also reported following emotion regulation training^81^ and relaxation training^80,82^. Others estimated session-by-session increases in self-rated decentering through intense repeated-measures designs. In adults with anxiety, EQ scores increased by a unit of 0.49–0.66 per session of CBT and 1.5 per four sessions of Acceptance and Commitment Therapy (ACT). Specifically, adults began treatment with EQ scores of around 29.32–34.99 (±6.12–6.17) and this grew by a unit of 0.37–0.66 per session (27.8–34.41 years)^70,71,82^. In adults seeking treatment for anxiety, self-rated cognitive fusion increased by a unit of 1.36 per CBT session and by 1.96 per ACT session (M baseline = 18.45–18.34) (37 ± 11.80 years). While these studies assume gradual changes in decentering across psychological interventions, there is also evidence of curvilinear growth during ACT and CBT^83^. This is characterized by an initial, rapid increase in decentering that flattens over time. Decentering thus is a sensitive psychological ability and gains may be quickly accrued during early stages of therapy.

**Table 2:** Summary of published studies that outline the impact of psychological interventions on decentering-relateda abilities in psychiatric and non-psychiatric samples.

Authors	Year	Sample characteristics	Psychological Intervention	Decentering-related ability	Decentering measure	Intervention stage	*M* age (years)	Impact of intervention on decentering-related abilities
Eustis et al.	2018	Healthy sample	ABBT	Decentering	EQ, AAQ	–	25	Decentering-related skills improved in ABBT.
Hayes-Skelton et al.	2015	Generalized anxiety disorder	ABBT, AR	Decentering	EQ	Treatment	34	Decentering-related skills improved in both conditions.
Gillanders et al.	2014	Probable case of minor psychiatric disorder	ACT	Cognitive defusion	CFQ	Treatment	41	Decentering-related skills improved in ACT but not comparison condition.
Scott et al.	2016	Pain-related distress and disability	ACT	Cognitive defusion	CFQ, EQ	Treatment	46	Decentering-related skills improved overtime.
Ostergard et al.	2020	Residual depression symptoms	ACT	Cognitive defusion	CFQ, AAQ	Relapse prevention	41	Decentering-related skills improved overtime.
Arch et al.	2012	One more more anxiety disorders	ACT, CBT	Cognitive defusion	BAFT	Treatment	37	Decentering-related skills improved in both conditions.
Forman et al.	2012	Anxiety/depression symptoms	ACT, CBT	Cognitive defusion	ATQ	Treatment	28	Non-linear improvement in decentering skills in both groups overtime.
Zettle et al.	2011	Moderate-severe depression	ACT, CBT (minus self-distancing)	Cognitive defusion	ATQ	Treatment	–	Decentering-related abilities improved in ACT but not CBT (minus self-distancing) condition.
Twohig et al.	2010	OCD	ACT, PRT	Cognitive defusion	AAQ	Treatment	37	Decentering-related skills improved in both conditions.
Hayes-Skelton et al.	2018	Social anxiety	CBT	Decentering	EQ, AAQ	Treatment	28	Decentering-related skills improved overtime.
Hayes-Skelton et al.	2019	Social anxiety	CBT (group-based)	Decentering	EQ	Treatment	28	Decentering-related skills improved overtime.
Teasdale et al.	2002	Residual depression symptoms	CBT, MBCT	Meta-cognitive awareness	MACAM	Relapse prevention	43	Decentering-related abilities improved in both conditions.
Frab et al.	2018	Remission from depression	CBT, MBCT	Decentering	EQ	Relapse prevention	40	Decentering-related skills improved in both conditions.
Segal et al.	2019	Remission from depression	CBT, MBCT	Decentering	EQ	Relapse prevention	40	Decentering-related abilities improved in both conditions.
O’Toole et al.	2019	Generalized anxiety disorder	ERT	Decentering	EQ	Treatment	22	Decentering-related skills improved overtime.
Hoge et al.	2015	Generalized anxiety disorder	MBSR, SM	Decentering	EQ	Treatment	37	Decentering-related skills improved overtime in the MT relative to comparison condition.
Orzech et al.	2009	Healthy sample	MT	Decentering	EQ	–	53	Decentering-related skills improved overtime in the MT relative to comparison condition.
Shoham et al.	2011	Healthy sample	MT	Decentering	Single item	–	26	Self-rated decentering improved during meditation practice.
Beiling et al.	2012	Depression	MT, medication	Decentering	EQ	Relapse prevention	42	Decentering-related skills improved in MBCT group
Josefsson et al.	2012	Healthy sample	MT, RT	Decentering	EQ	–	49	No change in decentering-related skills over time.

Only studies that included (at least) pre and post-intervention meausres of decentering-related abilities are presented.

*ACT* acceptance and commitment therapy, *CBT* cognitive behavioral therapy, *PRT* progressive relaxation training, *MBCT* mindfulness-based cognitive therpay, *ERT* emotion regulation training, *MBSR* mindfulness-based stress reduction, *SM* stress management, *MT* mindfulness trianing, *RT* relaxation training, *EQ* experiences questionnaire, *CFQ* cognitive fusion questionnaire, *AAQ* acceptance and action questionnaire, *BAFT* believability of anxious thoughts and feeling scale, *MACAM* measure of awareness and coping in autobiographical memory.

An important question is– how can different psychological interventions, each grounded in distinct philosophical and scientific traditions, commonly improve decentering? This is arguably because interventions employ techniques that have a shared intention to influence the way individuals relate to, and observe, day-to-day psychological stressors (like thoughts, feelings and memories). In doing so, these techniques recruit and refine the ability to adopt an objective awareness of negative inner events and their underlying cognitive activities. This is an explicit focus within third-generation treatments including ACT and Mindfulness-based Cognitive Therapy (MBCT)^12^. Techniques like the mindful monitoring of experiences and cognitive defusion explicitly undefined the content of negative inner events while encouraging a meta-awareness of activities such as the act of thinking, remembering, or mind-wandering^14,84–86^. This is reflected by evidence that the negative association between trait mindfulness and anxiety severity is partially mediated by decentering-related abilities^69,87^. Other approaches like CBT indirectly cultivate decentering via exercises that challenge the content of negative inner events. Techniques like cognitive restructuring or re-appraisal first prompt individuals to self-distance by taking an objective perspective and identifying distorted beliefs before testing their reliability^88^. Indeed, the salutary effects of re-appraisal strategies on anxiety may be mediated by decentering-related abilities^69,71,81^.

Psychological interventions that explicitly target decentering may shape this ability even better. Greater increases in decentering were found across ACT relative to CBT^73,89^ or relaxation training^80^. Although these findings are equivocal with others reporting non-significant effects of intervention-type on decentering^82,83^. Regardless, psychological interventions gradually reinforce decentering by targeting the way individuals relate to, or experience, negative inner events. At-home practice of MBCT and CBT skills in the weeks following treatment predicted increases in decentering over 24 months in adults in remission from depression (40.41 ± 11.61 years)^90^. It is difficult to explain the observed increases in decentering across psychological interventions through other parsimonious accounts. It could be, for instance, that increases in decentering simply reflect treatment-related decreases in anxiety/depression. Indeed, anti-depressant medication indirectly increased self-rated decentering in adults experiencing depression via decreases in depression severity^74^. Yet this study also reported continued gains in decentering in a sub-set of individuals who went on to complete MBCT and not those who continued with anti-depressant medication. This suggests that ameliorating symptom severity partially improves decentering tendencies but more substantial gains in this skill may be unique to participation in psychological interventions. However, few have examined treatment-related increases in decentering outside of psychological interventions. The specificity of this therapeutic component and the exact mechanism through which clinical changes take place therefore remain unclear.

### Increases in decentering mediate reductions in symptom severity

The reinforcement of decentering skills may be a common pathway through which different psychological interventions – like CBT, ACT, and MCBT, among others – commonly reduce anxiety and depression symptoms (Table 2). Treatment-related increases in decentering mediated the impact of mindfulness training on anxiety severity in adults seeking treatment for generalized anxiety disorder (GAD) (37.6 ± 11.6 years)^87^. In a similar group, the rate at which decentering increased across treatment was associated with overall decreases in worry (*r* = −0.74) and stress (*r* = −0.76; this study involved acceptance-based treatment and applied relaxation) (34.14 ± 12.14 years)^82^. Session-by-session increases in decentering also predicted treatment-related decreases in social anxiety in adults seeking treatment through CBT (27.90 ± 10.06 years)^70,71^. Within acceptance-based treatments, increases in cognitive defusion predicted decreases in depression symptoms and psycho-social dysfunction in adults experiencing pain-related distress (46.40 ± 11.6 years)^73,79^. Additionally, and in adults seeking treatment for multiple anxiety disorders, session-by-session increases in self-rated cognitive defusion mediated decreases in worry, depression, avoidance and psycho-social dysfunction – this was evident in both ACT and CBT^89^. Similar findings were reported in a transdiagnostic sample of young adults experiencing anxiety and mood-related disorders. Here, treatment-related increases in cognitive fusion mediated decreases in symptom intensity (25.40 ± 7.86 years)^83^.

Most studies cannot detect if decentering improves before symptoms decrease. This is a necessary criterion when establishing mediators of therapeutic change^91^. A small number of studies have therefore measured decentering and clinical outcomes more frequently – on a near session-by-session basis – to better infer a causal connection between decentering and clinical outcomes. In adults with GAD, a unidirectional relationship between improved decentering and reduced stress was identified during acceptance-based treatments and applied relaxation. This was characterized by decreases in stress occurring downstream to increased EQ scores (34.41 ± 12.14 years)^82^. A unidirectional relationship was also demonstrated in young adults seeking treatment for GAD via emotional regulation training (37.60 ± 11.70 years)^81^. Session-by-session increases in EQ scores were found to precede decrease in trait anxiety but not generalized anxiety symptoms. To our knowledge, there have been no attempts to chart the temporal relationship between increased decreased and reduced symptoms in standard interventions like CBT, ACT and MBCT. Future research ought to leverage high-resolution datasets to replicate and extend on these findings^92^. Nevertheless, emerging evidence suggests that early treatment-related increases in decentering facilitate subsequent improvements outcomes like anxiety, depression and psycho-social functioning.

### Decentering across different stages of mental health difficulties

The strengthening of decentering-related abilities may be a universally relevant component of psychological intervention. Techniques that target this skill could benefit those who are symptomatic, those who are experiencing remission and those who are even yet to develop mental health difficultie. This is because the ability to decenter mitigates the impact and distress associated with everyday psychological stressors experienced by most people.

In adults experiencing remission from depression, earlier relapse was predicted by baseline difficulties in decentering from recent negative memories (43.7 ± 9.60 years)^52^. Poorer decentering skills in this cohort were also associated with more severe residual symptoms of depression^4^. However, enhancing decentering-related abilities in the initial stages of remission can significantly reduce relapse risk. In a recent study with adults experiencing remission, ACT was associated with lower residual depression after 12 months and this was mediated by increases in cognitive defusion and psychological flexibility (40.77 ± 11.9 years)^78^. Decentering also increased in adults in remission who received follow-up care using MBCT and this predicted lower residual depression symptoms after 6 months (44.8 ± 9.4 years)^74^. Similarly, MBCT and CBT improved decentering in adults experiencing relapse from depression and this reduced relapse over a 24-month follow-up period (40.41–40.85 years)^90,93^. Interestingly, decreases in dysfunctional self-relevant beliefs during CBT (not MBCT) but this was unrelated to relapse risk^93^. This suggests that altering the way individuals experience negative inner events, rather than changing the content of these events, promotes sustained reductions in symptoms.

Few have investigated the effects of teaching decentering prior to anxiety or depression onset. However, there is some evidence of salutary effects in healthy individuals. Training of distancing techniques was associated with lower daily stress and a spontaneous tendency to provide a neutral evaluation of negative images (~23.9 years)^94^. Increased EQ scores also mediated the beneficial effects of mindfulness training on anxiety and low mood in non-symptomatic adults (49–54 years)^75,76^. Also, and in a cohort of university students experiencing elevated psychological distress, a brief acceptance-based program was associated with increased decentering and decreased stress and social anxiety (25.40 ± 7.86 years)^77^.

### Treatment based changes in youth decentering

One research gap is evident and particularly salient given this review’s focus. Few studies (if any) have investigated if increased decentering mediates the beneficial impact of psychological interventions in youth anxiety and depression. The mean age across the clinical trials described in this review (*k* = 20; Table 2) is 36 years and the youngest cohort we identified were around 25 years^77,83^. However, 75% of psychiatric disorders begin before 24 years^95^. The median age of onset for anxiety is around 11–15 years^95,96^, and 10% of adolescents begin to experience re-occurrent major depression before 17 years^97^. Childhood and adolescence is a well-established window for the development of mental health difficulties but is under-represented in this literature. Thus, it is not known: [1] whether psychological interventions reduce youth anxiety and depression via improvements in decentering, as observed across adulthood; or [2] whether reinforcing decentering via psychological interventions confers protection against the future onset of anxiety and depression in at-risk youths. These are key areas for future research, especially given that adolescents is a unique period of neuro-developmental change^98,99^ and findings based on adult research may not be directly generalizable.

A small number of recent studies described programs that reinforce decentering-related abilities during adolescence. The rationale is that teaching pre-symptomatic adolescents techniques to manage everyday psychological stressors may be an impactful way to promote well-being. Adolescents practiced a guided self-distancing technique over 10 days (18.47 ± 0.69 year)^100^. This involved recalling a stress-relevant event before taking a mental ‘step back’ to observe the event from a self-distanced perspective. Afterwards, participants wrote a short narrative about this experience. Fewer negative words and less ruminative processing was evident in the self-distanced group relative to a comparison group. However, there was no change in daily negative affect ratings in those practicing self-distancing. Also, it was not reported whether decentering-related abilities improved with practice. Another study examined a 30-min web-based cognitive defusion program for negative self-referential thinking in adolescents (19.61 ± 1.58 years)^101^. This resulted in large reductions in the believability of, and distress associated with, self-critical thoughts (*d* = 0.87–1.35). However, neither this study nor similar ones recorded changes in trait cognitive fusion or decentering^63,101–103^. It is therefore unclear: whether youth decentering-related abilities are sensitive to change or; whether decentering-related abilities can be improved using targeted training. Meanwhile in adults experiencing remission from depression, selectively teaching decentering resulted in higher trait decentering and lower residual symptoms (50.81 ± 12.10 years)^104^.

## Conclusion

### Whether, how, why, and when decentering alleviates youth anxiety and depression?

To try answer this question, we conducted a robust review of literature and our findings were as follows. Decentering is an early emergent ability that is continuously distributed across individuals. Those at the higher end of this continuum are at lower risk of anxiety and depression, across the lifespan. Evidence also suggests that decentering mitigates mental ill health by dampening the emotional impact of day-to-day psychological stressors that otherwise increase depression and anxiety risk: stressors include inner events such as distressing thoughts, feelings and memories. Better understanding this ability may therefore have implications for youth mental health and psychological interventions. In fact, evidence suggest decentering is a core component of psychological interventions whereby treatment-related improvements in this skill facilitate decreases in anxiety and depression severity. A range of extant psychological interventions (including CBT, ACT, and MBCT) have been shown to improve decentering-related abilities, and the magnitude of this improvement may mediate downstream decreases in anxiety and depression severity.

Furthermore, the reinforcement of decentering may be a universally relevant component of psychological interventions. This is because it targets day-to-day psychological stressors experienced by, not only those currently experiencing symptoms, but also those who may be pre-symptomatic or even recovering from depression or anxiety. This is supported by emerging evidence that reinforcement of decentering-related skills can be helpful at each multiple of mental health, including primary prevention, treatment and relapse prevention. Although a greater understanding of when and how best improve decentering-related abilities is required.

### Implications for practice and future research in youth anxiety and depression

Anxiety and depression are global health challenges with the majority of cases emerging before the age of 24 years^105,106^. This points to an urgent need to equip young people with strategies that help them influence the way psychological distress is experienced. In particular, we believe it is important to cultivate (from an early age) skills to adaptively manage everyday psychological stressors (e.g. distressing feeling, thoughts, memories), which involved in anxiety and depression onset and maintenance. We also believe that decentering is a promising candidate for three practical reasons. First, by shifting one’s self-perspective, people can still interact with psychological stressors without allowing them to disproportionately influence mood and behavior. This might help to, not only ameliorate distress in those experiencing symptoms, but also delimit youth anxiety and depression onset. Second, evidence suggests that ability to influence self-perspective can be strengthened with time and practice. This means it is a modifiable skill that can be directly reinforced. Finally, therapeutic techniques that boost decentering are already embedded within extant psychological interventions. It may be possible to isolate and amalgamate these modules so as to selectively train decentering in educational and care settings.

A number of research gaps must be addressed to fulfill the translational potential of decentering research (Supplemental Box 1). Among these is a lack of evidence indicating whether psychological interventions that reinforce decentering at an early age can delay anxiety and depression onset. The majority of clinical trials focus on symptomatic adults rather than pre-symptomatic young people. Another clear barrier is the paucity of child and adolescent-friendly measures of decentering-related abilities, as well as longitudinal investigations into the development of this skill. Finally, it is also important to identify the functional boundaries of decentering-related abilities in young people as well as the underlying neuro-cognitive correlates. Such insights have the potential to improve youth mental well-being, promote thriving across development and optimize the effectiveness of extant psychological interventions if and when they are needed.

## Supplementary information

Supplemental materials
