# Supplementary material for: Decentering as a core component in the psychological treatment and prevention of youth anxiety and depression: a narrative review and insight report
Source: Transl Psychiatry. 2021 May 14;11:288. doi: 10.1038/s41398-021-01397-5 (PMC8121888; doi:10.1038/s41398-021-01397-5)
Supplement: Supplementary file 1 — Supplemental materials [file 41398_2021_1397_MOESM1_ESM.docx]

**SUPPLEMENTAL MATERIALS**

**Decentering as a core component in the psychological treatment and prevention of youth anxiety and depression: A narrative review and insight report**

Marc P. Bennett^1*^ PhD,

Rachel Knight^1^ MSc,

Shivam Patel^1^ MSc,

Tierney Lee^1^,

Darren Dunning^1^ PhD,

Thorsten Barnhofer^2^ PhD,

Patrick Smith^3^ PhD,

Willem Kuyken^4^ PhD,

Tamsin Ford^5^ PhD,

Tim Dalgleish^1^ PhD,

^1^Medical Research Council Cognition and Brain Sciences Unit, University of Cambridge

^2^School of Psychology, University of Surrey

^3^ Institute of Psychiatry, Kings College London

^4^ Department of Psychiatry, University of Oxford

^5^Department of Psychiatry, University of Cambridge

*Correspondences should be addressed to Dr. Marc Bennett,15 Chaucer Rd, Cambridge CB2 7EF, United Kingdom

Marc.bennett@mrc-cbu.cam.ac.uk

### **How was this insight report prepared?**

**Literature review.** This report reviews the literature on decentering-related abilities with a special focus on its impact on youth anxiety and depression. We identified published, peer-reviewed papers from a range of study-types. This included: *cross-sectional studies* examining the association between decentering-related abilities and mental health (e.g. anxiety and depression severity): laboratory based *component studies* examining the impact of brief decentering inductions on affect (e.g. mood and emotional reactivity): and *clinical trials* examining the impact of psychological interventions for anxiety and depression on decentering-related abilities. The objectives were to: clarify and consolidate extant conceptualizations of decentering-related abilities (Introduction to decentering), characterise the association between decentering and affective outcomes (Decentering, development and mental health), and determine the effect of extant psychological interventions on decentering at different stages of mental health (Decentering and psychological intervention). Thus, this report may be best classed as a variant of a *scoping review*. This was an appropriate strategy given the paucity of previous reviews, and the diversity of constructs and study-types that we sought to integrate. Structured searches were carried out using an online database. Hits were screened for eligibility before being classified by study-type before extracting relevant details. A narrative synthesis was then conducted^1^. Research and clinical implications are summarized in Supplemental Box 1.

**Patient and Public Involvement.** A youth advisory panel was established to provide expert feedback and to help shape this report. Their role was: to examine if the current literature and its findings reflect the experiences of young people: and to identify avenues for future research by highlight gaps in our knowledge base. The youth advisory panel comprised of four individuals (14–25 years old) from a diversity of socio-economic backgrounds. All advisors had a special interest in mental health and at least two had a previous, personal experience with mental health difficulties. Youth advisors were recruited via a small-scale and strategic advertisement campaign that involved an open call for ‘young people with a special interest in mental health’. Youth advisors were consulted on a range of materials related to decentering including a brief introduction to decentering-related abilities, previously written blog posts and self-report inventories. Feedback was gathered via semi-structured focus groups and the information was synthesised by members of the research team. Paraphrased excerpts are presented and explored in Supplemental Boxes 2–5.

## **Review Methods**

**Inclusion criteria.** Findings were considered across three types of studies. [1] Cross-sectional studies: that is, studies charting patterns of co-variation between self-rated decentering-related abilities and mental health difficulties like anxiety and depression severity as well as self-rated quality-of-life. [2] Component studies: that is, laboratory-based experimental studies examine acute changes in affect due to the delivery of a brief decentering manipulation. This included, but was not limited to, studies that instructed individuals to self-distance/adopt a third-person perspective as well as studies that used brief cognitive defusion exercises. [3] Clinical trials: that is, studies that examine changes in decentering-related abilities and mental health outcomes due to the delivery of a psychological intervention. This included, but was not limited to, cognitive behavioural therapy (CBT), acceptance and commitment therapy (ACT), mindfulness-based cognitive therapy (MBCT) and applied relaxation (AR).

**Exclusion criteria.** The following exclusion criteria were applied to studies: n-of-1 designs and case studies, qualitative designs, Unpublished dissertations, encyclopaedia entries, conference abstracts and presentations, book reviews and chapters, letters and editorials, other meta-analysis and systematic reviews. Studies recruiting individuals with neurodevelopmental conditions will be excluded (e.g. autism and ADHD). Studies were also excluded if they did not include a measure of decentering-related abilities. It was also decided to focus on studies that documented the association between.

**Database and search term.** Searches were conducted in Web-of-science. The final search date was 17^th^ of July 2020. The search term was (TI = ("cognitive defusion" OR "self-as-context" OR self distanc* OR "distancing" OR "decentering" OR "decentring" OR "decentered self" OR "detached mindfulness" OR "self-distanced perspective" OR "meta-cognitive awareness" OR "meta-awareness" ) OR AB = (" cognitive defusion" OR "self-as-context" OR self distanc* OR "distancing" OR "decentering" OR "decentring" OR "decentered self" OR "detached mindfulness" OR "self-distanced perspective" OR "meta-cognitive awareness" OR "meta-awareness" ) AND (TI = (emotion* OR affect* OR mood OR anxiety OR depression ) OR AB = (emotion* OR affect* OR mood OR anxiety OR depression))).

**Participants details.** Given the paucity of clinical trials examining the impact of psychological interventions on child and adolescent decentering-skills, we placed no age restrictions. It was decided that findings would be grouped according to broad developmental cohorts, where possible. This included children (< 11 years), early adolescents, (11–16 years), older adolescents (17–20 years), emerging adults (21–24 years old) and adults (> 24 years). One aim was to examine whether decentering-related abilities are relevant across different stages of mental health difficulties. Therefore, we decided to include studies that recruited a range of individuals. This included individuals: [1] without any depression and anxiety symptoms; [2] at-risk of anxiety and depression; [3] experiencing anxiety and depression symptoms; and [4] individuals recovering from anxiety and depression or those experiencing relapse. Where possible, findings were considered separately for these groups.

**Extraction details.** Following the initial search, hits from different search engines were collated and then de-duplicated. Titles and abstracts were screened for relevance by at least two reviewers (MB & RK). This consolidated list of potential studies was then be agreed by the reviewers, at which stage full-text papers will be assessed against in terms of the reviews’ eligibility criteria. Screening and extraction were tracked using a combination of MATLAB scripts and excel spreadsheets- all of which will be made available on request to MB.

The following descriptive data were then be extracted from the eligible hits. These data were extracted by two independent reviewers (MB & RK).

- Population characteristics including age range (e.g. children, adolescents or adults), anxiety and depression symptom severity, diagnostic status (e.g. diagnosed, relapsed or remitted).
- Study characteristics including sample size, study-type (e.g. clinical trial, experimental analogue, or cross-sectional design).
- Decentering characteristics including mode of decentering (e.g. self-distancing, self-as-context, cognitive defusion), assessment of decentering (e.g. self-reported measures, clinician ratings or behavioral assessment).
- Intervention characteristics, where appropriate, including the type of psychological therapy (e.g. cognitive therapy, third generation therapy, or experimental analogue) and timing/structure of the intervention.
- Comparison characteristics, where appropriate. For example, a non-specific attentional control group or clinical management.
- Primary and secondary outcome characteristics including the instruments used to measure anxiety and depression symptoms and the time points when these experiences were sampled.
- Statistical methods (e.g. whether and what type of mediational analysis was calculated).
- Researcher characteristics, including the name(s) of the lead researchers, contact information and universities.

**Review process.** Two team members (MB & RK) conducted structured searches via Web-Of-Science (final search date = 17^th^ July 2020). We searched for titles/abstracts containing: the terms ‘decentering’, ‘decentring’, ‘decentered self’, ‘cognitive defusion’, ‘self-as-context’, ‘(self)-distancing (perspective)’, ‘detached mindfulness’ or ‘meta-cognitive awareness’: and the terms ‘emotion’ ‘affect’, ‘mood’, ‘anxiety’ or ‘depression’. This search revealed 1414 hits. We then excluded papers published in fields other than psychology, psychiatry or health (e.g. engineering, computer science, physics). This left 810 hits whose abstracts were screened based on pre-defined inclusion and exclusion criteria (e.g. relevant trial-type and including measures of decentering and negative affect or symptom severity). Afterwards, 151 manuscripts were subject to a full-text review where relevant information was extracted. This included: k = 52 cross-sectional studies (9 with children and adolescents [<18 years], 7 with emerging adults [< 24 years], and 35 adults [25 years]); k= 68 experimental studies (20 with children and adolescents, 20 with emerging adults, and 28 with adults); and k = 34 clinical trials (3 including adolescents and 31 with adults). The main report represents a narrative synthesis of the main findings across these papers. This synthesis was based on available guidance from the Economic and Social Research Council^1^.

### **Supplemental Box 1: Summary and recommendations**

Psychological interventions could deliver comparable reductions in symptom severity by boosting decentering. This skill emerges at an early age and it dampens the distress associated with day-to-day psychological stressors that otherwise increase the risk of anxiety and depression onset and maintenance. Thus, the strengthening of decentering is a pan-therapeutic mechanism and is also universally relevant across different stages of mental health difficulties.

**Research recommendations.** More information about the development of decentering-related abilities is needed. This knowledge can be leveraged to create techniques that strengthen decentering from an early age, thus improving how young people manage day-to-day psychological stressors. This could be an efficient and impactful way to promote mental well-being. This research also requires additional valid measures and methods to experimentally investigate decentering in young people.

**Practical recommendation.** It is important to explicitly cultivate an ability to shift awareness – from the negative content of an inner event towards the underlying cognitive activities. Techniques to achieve this outcome are already nested within psychological interventions (e.g. self-distancing and cognitive fusion). It can be helpful to target this pathway more directly and monitor changes decentering-related abilities, e.g. using brief self-report measures like the Experiences Questionnaire^4^ or even short, single-item questions^31^. This can have important implications on symptom reduction and relapse prevention.

**Policy recommendation.** Clinical science and practice will benefit from opportunities to further investigate the boundary conditions of active ingredients like decentering. This requires the support research programmes that will, in turn, provide high-impact technologies. This will include low-intensity and highly scalable prevention programmes that improve young people’s ability management of day-to-day stressors (Box 5). There are opportunities to deliver such programmes at low-cost by leveraging mobile technologies^104-106^. This also requires the creation of creative collaborative spaces that bring together experts from different fields (e.g. scientists, clinicians, developers, promotors and PPI advisors, to name a few).

### **Supplemental Box 2: Young peoples’ views on decentering and self-observation**

Youth advisors reported experience with maladaptive self-observation styles such as a tendency to overly-focus on the content of negative inner events (Excerpt 1). They were also practically familiar with the concept of decentering, and this was broadly endorsed as a helpful strategy (Excerpts 2-4). Advisors also implied that one’s social circle could help facilitate decentering (Excerpt 3). We are unaware of research examining this link.

***Excerpt 1:*** *You might have a thought, even as a joke, "I hate myself", or you say "I hate myself " more seriously. Even in saying that, you're saying "I hate myself" rather than saying "sometimes I have thoughts that make me think I hate myself". It’s easy to sort of embody yourself as the anxiety rather than it just being a feeling.*

***Excerpt 2:*** *When you’re in the present you seem to think that smaller things are a lot bigger than they are. You think everything is much more significant and will impact you a lot more than it actually does.*

***Excerpt 3:*** *I think when I’m worrying about things like school, it’s helpful to think of it from a friend’s perspective because you know your friends would want to help you through that. When you think of it like that it calms you down.*

***Excerpt* 4:** *Decentering would be good for people with mental health difficulties. Otherwise you’d only think about things from your perspective. You would just be stuck. It’s just you thinking in your brain- little things become bigger things because you think of things in terms of how you feel rather than how they actually are. When you take a step back and see it from another perspective, I think you’d see things more clearly.*

### **Supplemental Box 3: The development of decentering according to young people**

Youth advisors highlighted age as an important factor influencing the efficient use of decentering-related abilities (Excerpt 1). Specifically, decentering was thought to be easier with time and experience. It was also suggested that a fundamental awareness of inner events is necessary before one can decenter. It was highlighted that this level of awareness is not always easy (Excerpts 2-3). We were unaware of studies exploring a link between emotional awareness^108^ and decentering.

***Excerpt 1:*** *Noticing your thoughts and feelings can be hard to do as a teenager. When you’re older it can become easier to notice what you’re feeling.*

***Extract. 2:*** *You'd need to be aware of your feelings to use some decentering techniques, but this is something even very young people could use.*

***Excerpt 3:*** *Decentering can be hard to do if you're young or not in tune with yourself, but would be helpful, especially for people who aren't as in tune with themselves.*

### **Supplemental Box 4: The impact of decentering according to young people**

Research suggests that decentering reduces the impact and distress associated with day-to-day psychological stressors (e.g. negative thoughts, feelings and memories). This seems to reflect the experiences of young people (Excerpts 1–4). Youth advisors also highlighted that decentering also facilitate changes in affect by allowing individuals to reconstrue negative experiences in terms of the broader context; that is, helping one see the bigger picture^17,108^ (Excerpt 1, also Box 2).

***Excerpt 1:*** *It is easier to assess the situation outside of when it’s happening. It’s easier to think about because you can see things more logically and from a more reasonable perspective.*

***Excerpt 2:*** *When I’m worrying about things like school, it’s helpful to think of it from a friend’s perspective because you know your friends would want to help you through that. When you think of it like that, it calms you down. Decentering helps you learn to deal with little things and not turn them into something that is big.*

***Excerpt 3:*** *Even that initial exercise of taking a step back can really help. When you do actually notice your thoughts and feelings, it does help me. It silences that critic. If I can sort of say that thoughts are just thoughts and come back to reality, it is really helpful.*

***Excerpt 4:*** *I think it’s sort of helpful to get some kind of perspective on your situation because you can realise how almost inconsequential the things you are worrying about is in away.*

### **Supplemental Box 5: The strengthening of decentering according to young people**

Youth advisors endorsed training programmes that teach decentering-related abilities (Excerpt 1-2). It was suggested that teaching helpful ways to safely experience negative inner events might undermine day-to-day pressures to feel a particular way, e.g. only feeling positive. Such demands were thought to relate, in part, to the use of social media (Excerpt 3-5). Advisors also highlighted symptom severity as an important consideration for teaching methods; those with more severe symptoms could benefit from more direct training of decentering skills (Excerpt 6). Importantly, advisors noted a tension between creating new mental health programmes and increasing pressure on young people (Excerpt 7). This implies that accessibility and user-experience are vital components for future research.

***Excerpt 1:*** *Instead of keeping things in, when they just build up, it’s good to notice how you’re feeling and you figure it out and learn how to deal with it.*

***Excerpt 2:*** *I think this would be quite helpful for people who never tried to examine things. People who just say ‘that’s who I am’ or ‘that’s just the way things are’.*

***Excerpt 3:*** *I think there is an atmosphere of toxic positivity. It’s almost like as soon as you feel bad or sad about anything it’s like ‘whoa there’s something really wrong with you’. But no, things happen. You’re okay most of the time and you go up and you go down. I think most of us have a fear of being anxious now. It’s almost like you’re anxious about being anxious.*

***Excerpt 4:*** *I think it’s useful to normalise negative thoughts and feelings. Not fight them.*

***Excerpt 5:*** I think what causes that anxiety for young people is probably having to digest on social media. Its people are so happy and just living their lives and that can make me feel really anxious. I think the overall anxiety is I’m not I’m not living my best life

***Excerpt 6:*** *Speaking from the perspective of someone with anxiety, I think decentering would come easier for someone who experiences normal anxiety on a daily basis. I think what would be helpful for people with anxiety disorders would be if they are more directly trained to decenter. I think it’s more like you feel so out of control with your mind, you almost need to be trained on how to rein your mind in. I think it would be helpful if there was an actual person helping you to do it.*

***Excerpt 7:*** *With this new age of self-care and mindfulness, I’m like ‘god I even suck at that’ you know? It’s kind of another thing on the list that you’re supposed to be doing.*
